# Knowledge Mapping of Volunteer Motivation: A Bibliometric Analysis and Cross-Cultural Comparative Study

**DOI:** 10.3389/fpsyg.2022.883150

**Published:** 2022-06-03

**Authors:** Jing Chen, Chengliang Wang, Yulong Tang

**Affiliations:** College of Education, Zhejiang University of Technology, Hangzhou, China

**Keywords:** volunteer motivation, cross-cultural perspective, knowledge map, bibliometrics, VOSviewer, CiteSpace

## Abstract

Volunteers play an indispensable role in several major events and activities. The purpose of this study is to review studies on volunteer motivation from 2000 to 2021 and to discover the development trends in this field. The Web of Science Core Collection is the main literature data resource, from which 162 papers on volunteer motivation published in the SSCI were selected. Using two visualization analysis tools, CiteSpace and VOSviewer, this study conducts bibliometric analysis and systematic review from multiple dimensions, identifying the authors, countries, institutions, and journals with high productivity in this field. Additionally, we explored highly cited papers, authors, and journals in this field. This study aims to find the research hotspots and theoretical basis through co-occurrence analysis and cluster analysis of keywords and explore the evolution through the time zone map drawn with CiteSpace. Moreover, we focus on the influence of Chinese and Western cultures (represented by China and the United States) on volunteer motivation. It was found that Chinese volunteers were more affected by collectivism, whereas American volunteers were more affected by individualism. The conclusion of this study constructs a clear framework for research on volunteer motivation, which provides researchers with a deeper and thorough understanding of the connotation of volunteer motivation, providing guidance and support for future research in this field.

## Introduction

In the twenty-first century, with the development of globalization, major events and activities have been held frequently. To ensure the high quality of various activities, the recruitment scale of volunteers is also expanding. For example, the number of volunteers recruited for the 2004 Olympics in Athens was 45,000, but the number reached 80,000 for the 2008 and 2012 Olympics in Beijing and London, respectively (Ahn, 2018).

Volunteers play a vital role in major events from general assistance (such as the assembly and distribution of materials) to specific assistance, which requires certain skills (such as medical support), organizing activities, and maintaining the order of events (Angosto et al., 2021). The great economic benefit offered by volunteers through their efforts and time effectively reduces the organizational and operational costs for organizers (Allen and Bartle, 2014; Pestereva, 2015). Some scholars believe that without the participation of volunteers, many large activities may face the risk of bankruptcy (Cuskelly et al., 2006).

Volunteer services tend to be defined as the behavior in which volunteers unconditionally provide numerous services to people other than close relatives. However, this behavior contributes immensely to the organizers while also providing many benefits to the volunteers (Gallarza et al., 2013). For example, when volunteers engage in activities for the purpose of giving back to society, they can obtain spiritual satisfaction simultaneously (Ma and Draper, 2016). Some volunteers gained a sense of achievement by serving others (Kao et al., 2019). The sense of achievement has been a crucial factor in promoting voluntary work and is, in addition, a significant part of volunteer motivation.

Although great progress has been made in research on volunteer motivation, there remain disputes regarding the basic structure or dimensions of volunteer motivation (Angosto et al., 2021). Moreover, the influence of diverse cultural and social backgrounds on motivation remains unclear. Existing research lacks a systematic and comprehensive literature review on volunteer motivation. Bibliometrics provides scholars with objective quantitative information including authors, keywords, journals, countries, and institutions. This is the best research method to highlight the development, structure, frontiers, and evolution of a certain field (Abramo et al., 2011).

Meanwhile, with the development of the economy and society, international communication and cooperation are continuously increasing. Moreover, communication among distinct cultures has enjoyed a boom recently. Therefore, it is important to comprehend volunteer motivation from a cross-cultural perspective. Presently, many studies only analyze volunteer motivation in the background of Western culture, with a few cross-cultural studies. Some studies have compared the volunteering rate in different countries (Ruiter and De Graaf, 2006), while others have analyzed the differences between explicit and implicit motivations for volunteering in different nations (Aydinli et al., 2016). Therefore, it is necessary to systematically review existing research on volunteer motivation to learn its composition and structure from diverse cultural backgrounds. This study explored the following questions:

(1) From 2000 to 2021, which are the active authors, institutions, and countries in the research of volunteer motivation?
(2) From 2000 to 2021, which major journals have published articles on volunteer motivation?
(3) What are the focuses in the research of volunteers? What is the evolution trend of research hotspots from 2000 to 2021?
(4) Will Eastern and Western cultures represented by China and the United States significantly influence volunteer motivation? If so, what is the mechanism behind this impact?

## Literature Review

Research on volunteer motivation in social science has a long history, and systematic research dates back to the early 1980s. Researchers have built a two-factor model focused on volunteer motivation, dividing motivation into altruistic and egoistic (Frisch and Gerrard, 1981; Smith, 1981). Morrow-Howell and Mui (1989) considered altruism, social, and material motivation to establish a three-factor model. Furthermore, they collected information using questionnaires to explore volunteer motivation.

Since the 1990s, researchers have paid more attention to volunteer motivation. Canan and Goldberg-Glen (1991) pioneered using factor analysis to classify volunteer motivation. Omoto and Snyder (1995) took volunteers for AIDS patients as the research objects and introduced the structural equation model (SEM) into their research on volunteer motivation. Based on empirical research, five functions of volunteering were presented, which receptively corresponded to five motivations, known as expressions of values, understanding, personal growth, community care, and the enhancement of self-esteem. Later, Clary et al. (1998) systematically summarized previous studies, proposing six types of motivation: values, understanding, social, career, protection, and enhancement. In accordance with these motivations, they formulated the Volunteer Functions Inventory (VFI).

With the dawn of the twenty-first century, volunteers at large sports events (such as the Olympic Games, Winter Olympics, and FIFA World Cup) and international exchanges of science, technology, and culture (such as the World Expo and various academic conferences) have become the focus of research on volunteer motivation. Taking volunteers in the 2004 Athens Olympic Games as subjects, Georgiadis et al. (2006) analyzed why they were willing to provide time-consuming services without any material benefits. Liao et al. (2012) studied volunteers in the 2009 World Games and explored the values of their participation motivation, work satisfaction, and perception ability in large sports events. Wang and Wu (2014) found in a study of 2010 World Expo volunteers' motivation and satisfaction that value expression, professional orientation, and love for the World Expo were their main motivations. Simultaneously, this study proposes many practical suggestions for the management of voluntary services. Volunteers in the 2018 Pingchang Winter Olympic Games drew the attention of scholars. Setting this Olympic Games as the background, Ahn (2018) expanded the research field of volunteer motivation to recognition and rewards in sports organizations and discovered a connection between multiple volunteer motivation dimensions, providing practical recruitment suggestions for organizers and managers. After investigating volunteers for the 2023 European Games, Rozmiarek et al. (2021) concluded that volunteers' experience, instead of gender, residence, or occupation, significantly influenced motivation.

Meanwhile, scales for measuring volunteer motivation have begun to diversify, such as Bang and Chelladurai's Volunteer Motivations Scale for International Sporting Events (Bang and Chelladurai, 2003) and Giannoulakis' Olympic Volunteer Motivation Scale (Giannoulakis et al., 2008). The VMSISE is the most widely used volunteer motivation scale in sports events (Angosto et al., 2021). To facilitate these needs, Bang et al. (2008) revised it again, adding a new variable, “love of sport,” which was verified by a questionnaire survey of volunteers in the Athens Games. In addition to sports events, VMSISE has been applied to other volunteer activities. For instance, Wang and Wu (2014) applied the scale to the study of people's volunteer motivation at the 2010 Shanghai World Expo following minor adjustments. Additionally, Vinnicombe and Wu (2020) creatively used VMSISE to study volunteers of music festivals. These studies widened the application scope of this scale, enriched the connotation of variables, and provided a reference for adjustments to the volunteer scale. OVMS passed the empirical test in the research of Athens Games volunteers and was successively used by scholars in the following international games to study volunteer motivation. Bang et al. (2019) applied it to the study of the relationship between motivation and satisfaction of volunteers during the 2016 Rio Olympic Games.

In the new century, research on volunteer motivation has focused not only on the distinction among volunteers in various activities but also on the differences in the motivation of volunteers of all ages from diverse social and family backgrounds (Okun and Michel, 2006; Wollebæk et al., 2014). At the same time, the research studied on special groups of volunteers is also increasing, such as volunteers with disabilities (Dickson et al., 2017), volunteers for international activities (Meneghini, 2016), and volunteers for Civil Defense (Kehl et al., 2017). Besides, cross-border and cross-cultural research has also attracted the attention of scholars. As enterprises that could profit through cross-cultural management (Chin and Rowley, 2018), organizers with cross-cultural knowledge will undoubtedly recruit and manage volunteers effectively in international events because experiencing different cultures lead to a better understanding of volunteers' hearts. Moreover, some scholars pointed out through empirical research that volunteers with a cross-cultural perspective will have a stronger willingness of volunteering which drives them to become stable volunteers in the long term (Aydinli et al., 2016).

Through meta-analysis in cross-border studies, Allik and Realo (2004) found that in countries with high GDP, a long-history political system of liberal democracy, and Protestants as the majority, residents participate in volunteering activities more frequently. Aydinli et al. (2016) also conducted empirical research comparing Eastern and Western cultures. By issuing questionnaires in China, Germany, Turkey, and the United States, through structural equation modeling, they verified that the volunteer motivation model, including implicit and explicit motivation, has certain universality in distinct cultural backgrounds.

Culture plays a complex role in motivation to volunteer. In previous research, differences in voluntary activities in different countries were attributed to changes in the community and community-related variables, such as socio-cultural value (individualism and collectivism), socio-demographic and socio-economic features, or political characteristics (Aydinli et al., 2013). For example, China and the United States, are the two countries with a high proportion of volunteers (>60%) (Rochester et al., 2010). Studies on volunteer motivation have found that Chinese volunteers' motivation is often reflected in value expression, patriotism, and variables that are identified with specific collectivism (Wang and Wu, 2014; Guo et al., 2021). However, empirical research on American volunteers showed that, in addition to community-service motivation, variables with individualism, such as personal career development and interests, are equally important components of volunteer motivation (Yamashita et al., 2019). Moreover, there are many studies focusing on the influence of religious beliefs (Allison et al., 2002; Mencken and Fitz, 2013; Cnaan et al., 2017). Scholars have conducted in-depth research on the mechanism by which different cultures influence motivation. Kagitcibasi (2017) believed that social economy, demography, and culture foster different lifestyles. In a society with more traditional norms, motivation influenced by collectivism is irresistible; while in a richer and more liberalized society, motivation conducive to personal growth becomes more dominant due to less reliance on society. Chin et al. (2021) highlighted that China has a group-centered collectivist culture, that encourages people to follow the rules formulated by the government for public interests. Undoubtedly, this would significantly influence the motivation of Chinese volunteers to participate.

The classification and connotation of volunteer motivation have also been complemented with a wider scope of the research field and continuous changes. For instance, owing to the impact of COVID-19, the sense of community responsibility has become an important source of motivation (Toubøl et al., 2022). Kifle Mekonen and Adarkwah (2021) studied the motivation of an exclusive group of postgraduate international students in China who participated in voluntary services during the COVID-19 crisis. Scholars have found that altruistic motivation is more prominent than ordinary motivation.

Based on the above discussion, it can be inferred that volunteer motivation tends to be affected by many personalized factors, such as region, national values, activity content, and cultural background. Obvious differences in volunteer activities can be seen in different eras, making the enduring topic of volunteer motivation worth studying.

## Methodology and Materials

### Bibliometric

Bibliometrics is a measurement method used to describe and analyze the situation and progress of a discipline or research field (Van Raan, 2019). According to Noyons et al. (1999), performance analysis and knowledge maps are the two major procedures in bibliometric analysis. Performance analysis is the performance and scientific productivity of authors, countries, and institutions that publish papers (Thelwall, 2008). In addition, the average citations per paper is a crucial index in performance analysis, which represents the influence of papers, authors, journals, institutions, and countries. Therefore, this quantitative index was added to the performance analysis in this study (Ding and Yang, 2020).

Knowledge maps are another valuable tool of bibliometric analysis which are often used to explore the framework structure and evolutionary path of key research fields (Cobo et al., 2011). With the help of modern computer technology, visual knowledge maps can clearly present the relationships among various elements in one research field (Merigó et al., 2015).

Knowledge maps can be divided into co-occurrence and evolution analyses. Keyword co-occurrence describes the situation in which two keywords appear in the same article; the more frequent the co-occurrence, the closer the connection of keywords, and so is the relationship. Through co-occurrence analysis of keywords, hotspots in a research field can be identified. Through evolutionary analysis, the development process and evolution trend of a research field can be learned (Ding and Yang, 2020; Baminiwatta and Solangaarachchi, 2021).

The two types of software VOSviewer (Version:1.6.17, developed by Van Eck and Waltman at the Centre for Science and Technology Studies) and CiteSpace (Version:5.8.R3, developed by Chen C. at Drexel University) were applied to draw a knowledge map. These two types of software are popular among many scholars in knowledge mapping. Through the analysis of 481 articles in bibliometric journals, Pan et al. (2018) found that CiteSpace is the most frequently used bibliometric software, although VOSviewer has been used often in recent years. Since the two types of software are based on distinct data analysis methods, they have different advantages. VOSviewer adopts a data normalization method based on probability theory. It can be used to construct a variety of visual maps of keywords, co-organizations, and co-authors. VOSviewer is simple to use, and its maps are clear (Van Eck and Waltman, 2010). In this study, VOSviewer was used to analyze keyword co-occurrence. CiteSpace adopts a data normalization method based on set theory to measure the similarity of data units from which the time zone view is drawn. Thus, the process of evolution and replacement of research hotspots in the dimension of time is evident (Chen, 2006). Therefore, researchers can learn the development process and trend of this field to conduct the evolution analysis of the research field explored in this paper.

Bibliometrics has been widely used in literature analyses since its independence as a discipline in 1969. This method, which reviews and investigates the existing literature in each field through quantitative methods, helps to generate a convincing conclusion (Mayr and Scharnhorst, 2015). In recent years, owing to the progress of different techniques (including but not limited to computer science, database management, and statistics), bibliometrics has been considered scientific and effective by an increasing number of scholars. Undoubtedly, bibliometrics has become a strong discipline for analyzing literature.

### Review Materials

This study chose the SSCI from the Web of Science (core collection) as the data source. The main reasons for this are as follows:

(1) As a high-quality digital literature database, Web of Science has been accepted by many researchers as the most suitable for bibliometric analysis (Ding and Yang, 2020). Compared with other databases, Web of Science provides data structures, including titles, authors, institutions, countries, abstracts, keywords, references, citation counts, impact factors, and others, including the data form that fits VOSviewer and CiteSpace (Carvalho et al., 2013; Gaviria-Marin et al., 2019).
(2) The topic of volunteer motivation belongs to social science, and previous studies also chose SSCI as the database (Jenkinson et al., 2013). Therefore, it is reasonable to choose articles from SSCI.
(3) There may be some interference factors if we introduce the database of SCI because some research, such as traditional Chinese medicine research use the word “volunteer” in their experiments, leading to disturbance of data.

The retrieval strategy this study adapted is TS = [(“volunteer” OR “volunteering”) AND (“motivation” OR “motives”)]. The period was from January 2000 to December 2021, and the deadline was December 31, 2021. Regarding the type of literature, we selected articles. A total of 472 journal papers were selected in the first step; a summary of the preliminary data is presented in Table 1.

**Table 1:** Summary of data source and selection.

**Category**	**Specific standard requirements**
Research database	Web of Science core collection
Citation indexes	SSCI
Searching period	January 2000 to January 22nd 2022
Language	“English”
Searching keywords	(“volunteer” OR “volunteering”) AND (“motivation” OR “motives”)
Subject categories	“Management” or “Sociology” or “Psychology”
Document types	“Articles”
Data extraction	Export with full records and cited references in plain text format
Sample size	475

If the information obtained from the database is used without careful screening, there may be problems such as repetition, deficiency, or inconsistency with the subject. Therefore, it is necessary to screen and standardize the data before analysis to avoid affecting the results and reducing inaccuracies owing to the quality of the data itself. According to the data standardization process proposed by Taskin and Al (2019), the steps of the data review and screening process in this study were as follows:

(1) Repeatability of the data was examined using CiteSpace. Consequently, four articles were excluded. Based on the literature screening criteria proposed by Su et al. (2020) and Jia et al. (2022), three team members independently examined the papers to screen out the articles that were inconsistent with the research topic. These controversial papers would be voted by our team to decide whether to be eliminated. After screening, 307 articles inconsistent with the subject were removed and 162 articles were retained.
(2) The author (AU) and source (SO) of the selected literature were corrected and unified. To avoid the impact of the names of authors and journals on results, we identified the authors of the same name and checked whether the journals to which the paper belongs have changed their names in the last 20 years.
(3) The keywords (DE and ID) were standardized. Non-standardized keywords will result in meaningless repetition in the keyword co-occurrence map because of the inconsistency of the parts of speech and the plural and singular versions. Therefore, all keywords must be simplified for analysis. The standard followed in this study is that for synonyms, low-frequency keywords should be unified as synonymous with high-frequency keywords. When the frequencies are similar, nouns should be prioritized. For example, for volunteer motivation, motivations, and motivation, the three keywords were unified as motivation in this study.

## Result and Discussion

**Figure 1:**
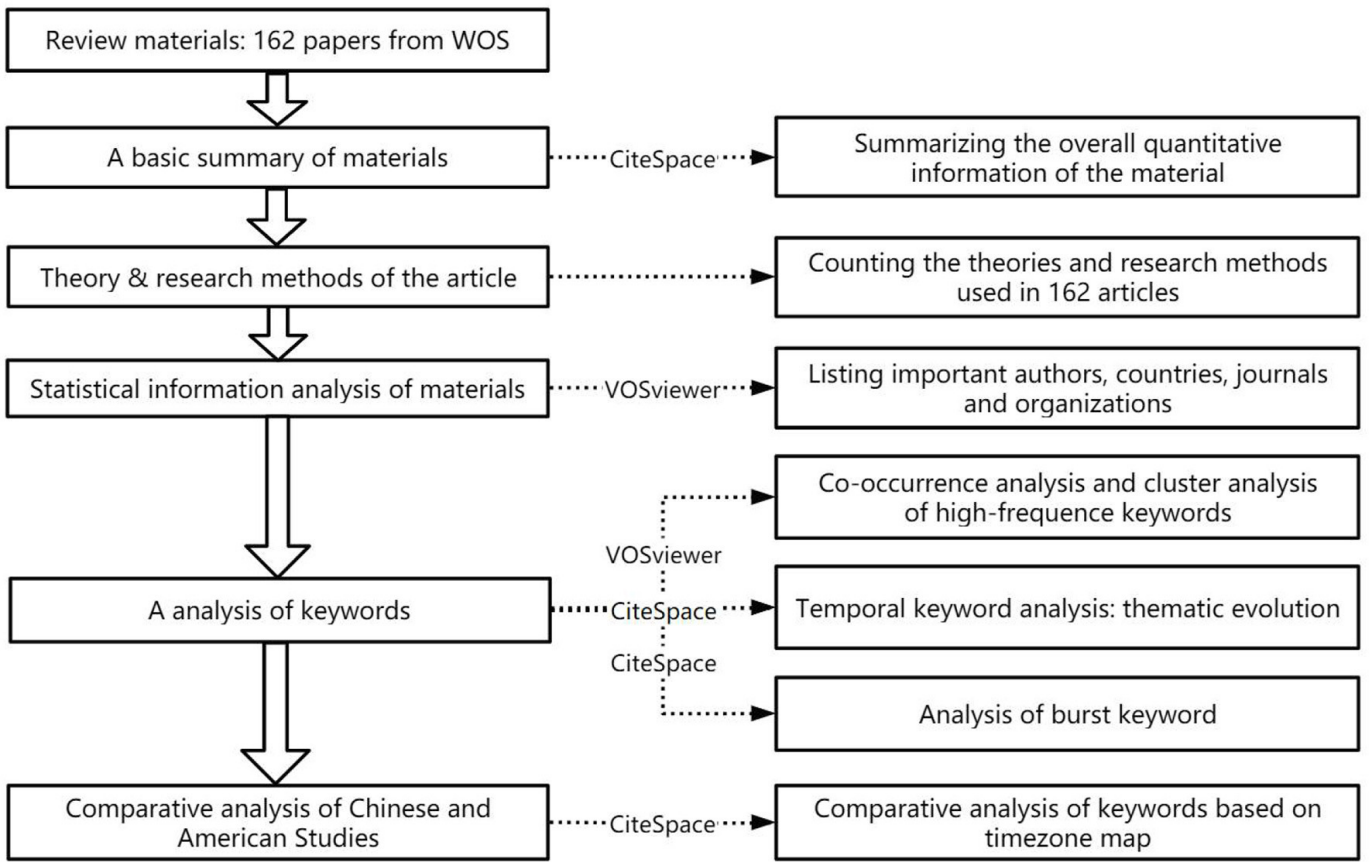
Analytical flowchart.

This section presents the descriptive statistics of authors, journals, and countries in the research field of volunteer motivation and the co-occurrence of keywords by the knowledge map and time zone map. In addition, we compared the research on Chinese and American volunteers, the results of which are shown in this section. An analytical flowchart of this section is shown in Figure 1.

### Summary of Data

The 162 papers analyzed in this study were written by 451 authors from 240 institutions in 40 countries, published in 77 journals, and cited 5,498 references from 2,724 publications (see Table 2).

**Table 2:** Summary of descriptive statistics.

**Criteria**	**Quantity**
Publications	163
Authors	452
Journals	77
Organizations	240
Countries	40
Cited reference	5,514

**Figure 2:**
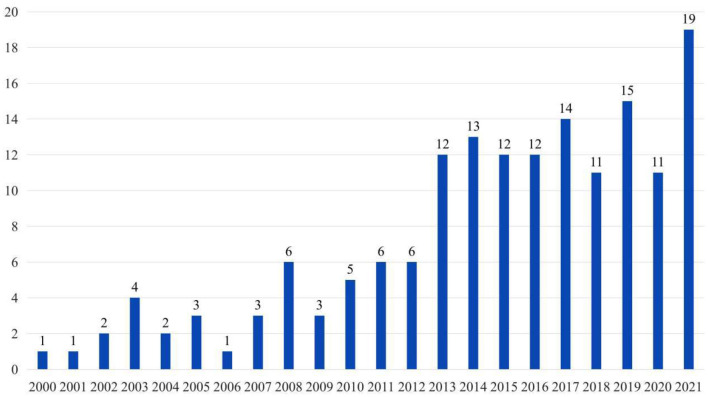
Time trend of publications on volunteer motivation.

Figure 2 reveals the distribution of publication times in the field of volunteer motivation. Overall, the number of studies on volunteer motivation is increasing and can be divided into two stages bounded by 2012. The first stage lasted from 2000 to 2012. Few articles were published every year, but the number of articles increased with continuous fluctuations. The second stage began in 2013 with a significant increase in the number of published papers. From 2013 to 2021, the average number of published articles exceeded 10. In 2021, this number reached 19, indicating that volunteer motivation has become more popular among scholars, and this field has ushered in vigorous development.

### Quantitative Analysis of Theoretical Basis and Analysis Method

These theories are of great significance in guiding research. According to the statistics of the 162 papers, 15 different theories were used. The number of studies in which each theory was adopted are presented in Table 3. Moreover, of the 162 studies, 15 used more than one theory, whereas 72 did not specify any theory. Of all these theories, functional theory and functional approach were those most frequently adopted by researchers to analyze volunteers' participation motivation (the two were applied in 45 studies), followed by self-determination theory (Deci and Ryan, 1985) and theory of planned behavior (Ajzen, 1985), which were applied in 25 and 16 studies, respectively. Other theories are role identity theory (Finkelstein and Brannick, 2007; Finkelstein, 2008, 2009; Marta et al., 2014), socioemotional selectivity theory (Okun and Schultz, 2003; Aydinli et al., 2015; Kulik, 2017; Yamashita et al., 2019), social cognitive theory (Wang et al., 2011; Hallmann and Zehrer, 2016), social exchange theory (Bang et al., 2019), and others (see Table 3).

**Table 3:** Theory used in the study.

**Theoretical framework**	***N***
No specific theory	72
Functional theory or functional approach	45
Self-determination theory	25
Theory of planned behavior	16
Role identity theory	4
Socioemotional selectivity theory	4
Social cognitive theory	2
Social exchange theory	1
Temporal self-regulation theory (Mullan et al., 2021)	1
Self-identity theory (Choi et al., 2020)	1
Theory of sport fan involvement (Koutrou, 2018)	1
Push and pull framework (Qi et al., 2019)	1
Social role theory (Hallmann et al., 2020)	1
Social cognitive career theory (Kao et al., 2020)	1
Structuration theory (Zanin et al., 2021)	1
Psychological contract theory (Haski-Leventhal et al., 2020)	1

Research methods refer to the tools and methods used to discover new phenomena and new things or to propose new theories and new points to reveal the internal laws of things. Among the 162 empirical research papers analyzed in this study, 17 studies used qualitative research methods, while 148 studies used quantitative research methods, 3 of which used both quantitative and qualitative methods (Pajo and Lee, 2011; Hyde and Knowles, 2013; Van Schie et al., 2015).

Interviews are the most commonly used method in qualitative research. Many interview studies use grounded theory to encode and analyze the information collected (MacNeela and Gannon, 2014; Williamson et al., 2018). Regression analysis and structural equation model (SEM) are the most frequently used methods in quantitative analysis. SEM includes factor analysis and path analysis. To clearly show the statistical results, this study stipulates that the research method be a single-row structural equation model and that the statistics not be repeated in the factor analysis and path analysis. Factor analysis (including exploratory factor analysis and confirmatory factor analysis) and correlation analysis (including *t*-tests and analysis of variance) are also widely used. Simple path analysis, which was used in 10 studies, was not popular. Four studies only made use of the descriptive statistics of the data (Gerstein et al., 2004; Pajo and Lee, 2011; Hyde et al., 2016; Dyson et al., 2021). The frequencies of the methods used are listed in Table 4.

**Table 4:** Research methods used in the study.

**Research methods**	***N***
Regression analysis	59
Structural equation model (SEM)	29
Factor analysis (Including EFA and CFA)	28
Correlation analysis	27
Path analysis	10
Descriptive statistics only	4

### Quantitative Analysis of the Author

Through the analysis of the number of papers published by each author, we can identify the representative scholars and the core research strength in the field of volunteer motivation. Price (1963), a scholar in the field of bibliometrics, indicated that for a research topic, half of the papers were written by a group of productive authors, the number of which is equal to the square root of the total number of authors. That is:

$$\begin{array}{l} \overset{I}{\underset{m+1}{\sum }}n\left(x\right)=\sqrt{N} \end{array}$$

where *n*(*x*) represents the number of authors who have written papers. I = *n*_max_ is the number of papers by the most productive author in this field (according to the statistics by VOSviewer, *n*_max_ = 8). N is the number of authors and m is the minimum number of papers published by the core author. According to Price's law, m = 0.749${}^{*}\sqrt{n_{\max}}$ ≈ 2.12. Therefore, authors with more than two articles (excluding two) were considered core authors in this field (Price, 1963). As a result, there were a total of 12 core authors. Table 5 presents the relevant information of all the core authors in this field, including their names, number of documents, and average citations per paper.

**Table 5:** Most important authors in the volunteer motivation research field.

**Rank**	**Author**	**Documents**	**Citations**	**Average citation per paper**
1	Wehner T	8	182	22.75
2	Okun M	4	501	125.25
3	Guentert S. T	4	111	27.75
4	Oostlander J	4	91	22.75
5	Van Schie S	4	85	21.25
6	Finkelstein M. A	3	174	58
7	Brudney J. I	3	135	45
8	Pepermans R	3	130	43.33
9	Guentert S. T	3	50	16.67
10	Li C	3	47	15.67
11	Bender M	3	44	14.67
12	Hallmann K	3	34	11.33

The research focus of different scholars varies significantly. Wehner has been the most productive author in the field of volunteer motivation in the past 20 years. He has published eight papers. He focused on empirical research using a structural equation model under the guidance of a functional approach, self-determination theory, or the theory of planned behavior. Okun is a renowned scholar of volunteer research. In the past 20 years, he has published four papers on volunteer motivation, which have been cited 125.25 times per paper on average. His main research subjects were senior-citizen volunteers. He focuses on the relationship between the change of ages and volunteer motivation (Okun and Schultz, 2003; Okun and Michel, 2006). In addition, he focuses on the impact of religious beliefs on volunteer motivation (Okun et al., 2015).

### Quantitative Analysis of Countries and Organizations

To identify the countries that have contributed the most in the field of volunteer motivation, this study analyzed the number of published papers from 40 countries. Table 6 lists the top ten countries in this field. According to the data in Table 6, the United States is the most influential country, with 49 papers (2,222 citations), accounting for 30% of the total. China and Australia ranked second and third, with 28 (353 citations) and 16 papers (626 citations), respectively. From the analysis of geographical distribution, it was found that most of the top ten countries are in North America and Europe, except China, Australia, and South Korea.

**Table 6:** Top 10 countries in the volunteer motivation research field.

**Rank**	**Country**	**Documents**	**Citations**	**Average citation per paper**
1	The United States	49	2,222	45.35
2	China	28	353	12.61
3	Australia	16	626	39.13
4	Switzerland	14	223	15.93
5	England	11	290	26.36
6	Germany	11	102	9.27
7	South Korea	10	102	10.2
8	Italy	9	151	16.78
9	Belgium	8	324	40.5
10	Spain	6	30	5

We further analyzed the publishing organizations. Table 7 lists the top ten institutions with the number of published papers. Among them, Arizona State University (the United States) and the Swiss Federal Institute of Technology (Switzerland) have the most papers in the field of volunteer motivation research, each has seven published articles. In addition, among the top 10, five institutions are in America, and their citations per paper are over 30. Arizona State University not only has the highest scientific productivity but additionally ranks the highest in terms of citations per paper. To some extent, these data reflect those institutions in America are in a leading position in this field.

**Table 7:** Top 10 influential organizations in volunteer motivation research.

**Rank**	**Organizations**	**Country**	**Documents**	**Citations**	**Average citation per paper**
1	Arizona State University	The United States	7	565	80.71
2	Swiss Federal Institute of Technology	Switzerland	7	119	17
3	Vrije Universiteit Brussel	Belgium	5	209	41.8
4	University of Queensland	Australia	5	189	37.8
5	University of Florida	The United States	4	267	66.75
6	The Hebrew University of Jerusalem	The United States	3	198	66
7	La Trobe University	Australia	3	196	65.33
8	University of Minnesota System	The United States	3	137	45.67
9	University of Pennsylvania	The United States	3	97	32.33
10	Griffith University	Australia	3	95	31.67

### Quantitative Analysis of Journals

Journals are important vehicles for publishing high-quality papers. Some scholars believe that there are two critical factors in evaluating journals' impact in certain fields: the number of papers published in the journals and the number of papers cited. The more published the articles, the more frequently their papers are cited, and the greater their impact in a certain field (Dzikowski, 2018). Therefore, our research analyzed ten journals on volunteer motivation, with the largest number of published papers. Simultaneously, we calculated the average citations per paper (Table 8).

**Table 8:** Top 10 journals in the volunteer motivation research field.

**Rank**	**Source**	**Documents**	**Citations**	**Average citation per paper**
1	Voluntas	24	301	12.54
2	Nonprofit and Voluntary Sector Quarterly	22	710	32.27
3	Sustainability	6	63	10.5
4	Journal of Social Service Research	6	50	8.33
5	Personality and Individual Differences	4	351	87.75
6	Social Behavior and Personality	4	223	55.75
7	Journal of Community & Applied Social Psychology	4	111	27.75
8	Journal of Social Psychology	4	75	18.75
9	Asia Pacific Journal of Tourism Research	4	19	4.75
10	Sage Open	3	11	3.67

Table 8 reveals that *Voluntas* (24 documents) and *Non-profit and Voluntary Sector Quarterly* (22 documents) ranked highest in the number of papers. This shows that the two journals focused on volunteer motivation. Other journals that published more than five pieces were *Sustainability* and *Journal of Social Service Research. Journal of Social Service Research* is a representative journal in the era of open access. In recent years, the rapid development of open-access journals has promoted research in this field. Although scholars have diverged on the best approach to open the source, the idea of sharing research achievements has reached a global consensus (Huang et al., 2020).

With further analysis of the average citations per paper in Table 8, *Personality and Individual Differences* are cited most frequently with 87.75 times per paper. The second was *Social Behavior and Personality* with 55.75 times per paper. Another journal with more than 30 citations is *Non-profit and Voluntary Sector Quarterly* (32.27 times per paper). This indicates that the documents published in the above 3 journals have gained more attention in research on volunteer motivation.

### Co-occurrence Analysis on Keywords

The keywords of a paper represent the focus of a study, while the keywords with high frequency illustrate the hotspots in a certain field. Cluster analysis of high-frequency keywords can show the macro situation of one research field. Wei et al. (2019) proposed that Price's Law can be applied not simply to analyze core authors in a certain field, but also to locate high-frequency keywords. According to the formula, M = 0.749${}^{*}\sqrt{\;N_{\max}}$ (N_max_ is the keyword with the highest frequency. According to VOSviewer, N_max_ = 112), M ≈ 7.92. Therefore, keywords with an occurrence frequency ≥7 are high-frequency keywords in this field.

**Figure 3:**
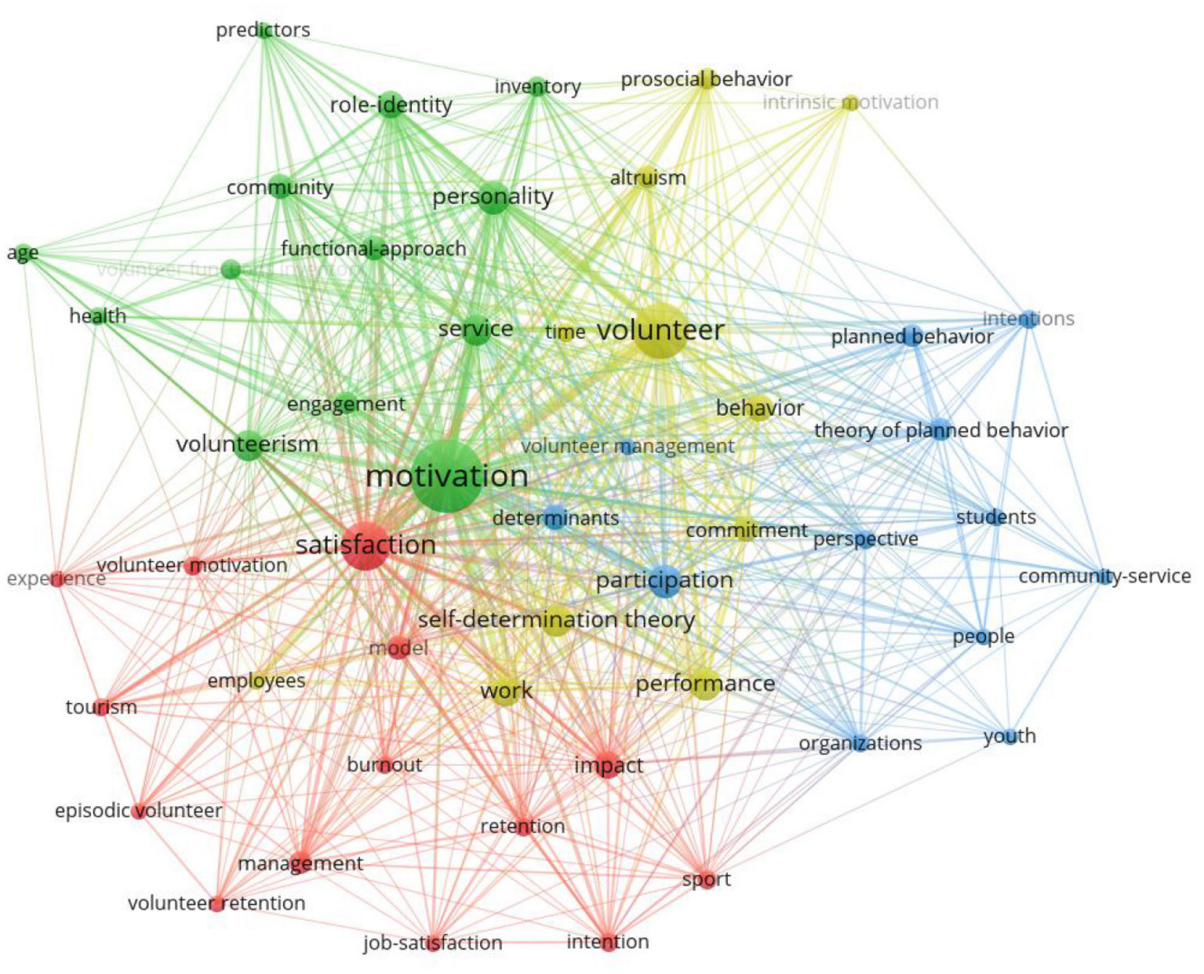
Co-occurrence of keywords.

The results of the visual analysis of the high-frequency keywords are shown in Figure 3. In the figure of the co-occurrence of keywords, the size of the node represents the occurrence frequency of keywords. The larger the node, the more frequently one keyword appears. Node colors represent different clusters or research topics: the line of nodes represents various levels of correlation intention, and the thicker the line, the more times the two appear together in the same literature.

Figure 3 depicts that yellow and blue clusters are closely related, whereas green and red clusters are relatively independent. To understand the correlation between keywords, our study sorted the vocabulary of the four clusters, as shown in Table 9.

**Table 9:** Cluster of keywords in the volunteer motivation research field.

**Cluster**	**Color**	**Keywords**
1	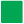	Functional-approach, age, community, engagement, health, motivation, personality, predictors, role-identity, service, volunteer functions inventory, volunteerism
2	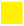	Self-determination theory, altruism, behavior, commitment, employee, intrinsic motivation, performance, prosocial behavior, time, volunteer, work
3	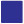	Theory of planned behavior, community-service, determinants, impact, intentions, organizations, participate, people, perspective, planned behavior, students, volunteer management, youth
4	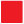	Satisfaction, episodic volunteer, burnout, experience, job satisfaction, management, model, retention, sport, tourism

The mainstream research spots in one field can be obtained through the analysis of high-frequency keyword clusters, which helps researchers understand more details within each focus. Next, the four clusters were analyzed sequentially.

The green cluster (cluster #1) of keywords centers on the functional approach (an approach to analyzing the structure and function of things and phenomena). The Volunteer Function Inventory (VFI) is devised under the guidance of functionalist theory (Clary et al., 1998). Most studies closely related to the green cluster of keywords choose research variables and organize a research framework based on VFI (Clary and Snyder, 1999; Allison et al., 2002; Finkelstein et al., 2005; Houle et al., 2005; Liao-Troth, 2005; Mowen and Sujan, 2005; Kim et al., 2010; Willems et al., 2012; Bang et al., 2013; Oostlander et al., 2014; Alexander et al., 2015; Khalemsky et al., 2020). Moreover, papers related to the green cluster of keywords combine functionalist theory and role identity theory to analyze the service motivation of volunteers. This indicates that the important service motivation is the role identity volunteers gain in social relationships in the organization (Penner, 2002).

The yellow cluster (cluster #2) of keywords is centered on the Self-Determination Theory (SDT). SDT emphasizes the subjective impact of people's egos on the formation of motivation. It identified that behaviors are formed based on psychological satisfaction (Deci and Ryan, 1985). The motivations for SDT fall into self-determined(intrinsic) and non-self-determined(extrinsic). Thus, there is egoistic and altruistic motivation. Papers related to the keywords in the yellow cluster were mostly written under the guidance of SDT. For instance, Finkelstein (2009) studied self-determination in volunteer services and found that intrinsic motivation is only one of the important aspects of self-determination, whereas extrinsic motivation is equally important. Meanwhile, in voluntary service, doing something meaningful is also one of the principal factors of volunteer motivation. Güntert and Wehner (2015) used empirical research methods to analyze the relationship between self-determination, controlling motivation, and volunteer identities. A significant causal relationship was found between self-determination motivation and volunteer identity. Van Schie et al. (2015) distinguished two focuses of self-determination motivation: general and organization-centered. Through empirical research, it was found that general self-determination motivation is related to the motivational potential of the task, whereas value consistency explains organization-centered self-determination motivation.

The keywords of the blue cluster (cluster #3) center on the theory of planned behavior (TPB). It focuses on the relationships among the variables of perceived behavioral control, subjective norms, attitude, volunteer motivation, and behavioral intentions (Kao et al., 2019). TPB was first proposed by Ajzen in 1985 and after its introduction into the field of volunteer research, immediately became a common theory to explain the motivation and intention of volunteers. For example, Warburton and Terry (2000) found that voluntary service motivation is jointly determined by social norms, perceived behavioral control, and moral obligations; Greenslade and White (2005) concluded that TPB could reasonably explain the high participation of the elderly in voluntary activities in Australia; Hyde and Knowles (2013) found through empirical research that TPB explains most of the variance in Australian college students' voluntary service intention. The study suggests that perceptual control and moral obligation should be used to encourage students to participate in voluntary activities. In addition, TPB is used to study the willingness of volunteers to stay on and predict the possibility of participation in future volunteer activities. Several studies have provided reasonable answers to volunteers' lasting motivation from the perspective of TPB (White et al., 2017; Almas et al., 2020).

The red cluster (cluster #4) focused on satisfaction. Relevant articles have primarily analyzed the relationship between volunteer motivation and satisfaction, such as the relationship between volunteer experience and future motivation for voluntary service (Bang et al., 2019; Kim et al., 2019). Other research studies the differences in volunteers' experiences motivated by several factors and found that volunteers with autonomous motivation have lower burnout than other volunteers (Ramos et al., 2016; Morse et al., 2020). Volunteer burnout is also an important keyword in the red cluster. After its proposal (Haski-Leventhal and Bargal, 2008) during the analysis of volunteer service stages of Israeli volunteers in 2009, volunteer burnout has been studied as a factor that influences volunteer motivation and leads to the loss of volunteers (Willems et al., 2012).

In conclusion, through cluster analysis, it is evident that theory is of great significance to the study of volunteer motivation, and an excellent research cannot succeed without corresponding theoretical guidance. Based on the clusters of keywords, we can further learn the core theories in volunteer motivation research and the focuses under the guidance of these theories. At the same time, based on Table 3, there are also some theories applied to volunteer motivation for less time. The purpose of using theories is often to find a reasonable explanation of volunteer service motivation and willingness from different perspectives. The continuous influx of different theories provides new visual aspects for volunteer motivation research, being an important boost to promote the development of this field.

### Temporal Keyword Analysis: Thematic Evolution

To identify the changes in research topics in different periods, which is a dynamic process, this study divides the timeline into two periods (the first from January 2001 to December 2010 and the second from January 2011 to December 2021). We analyze the keyword networks of the two periods to study the evolution of volunteer motivation research topics.

**Figure 4:**
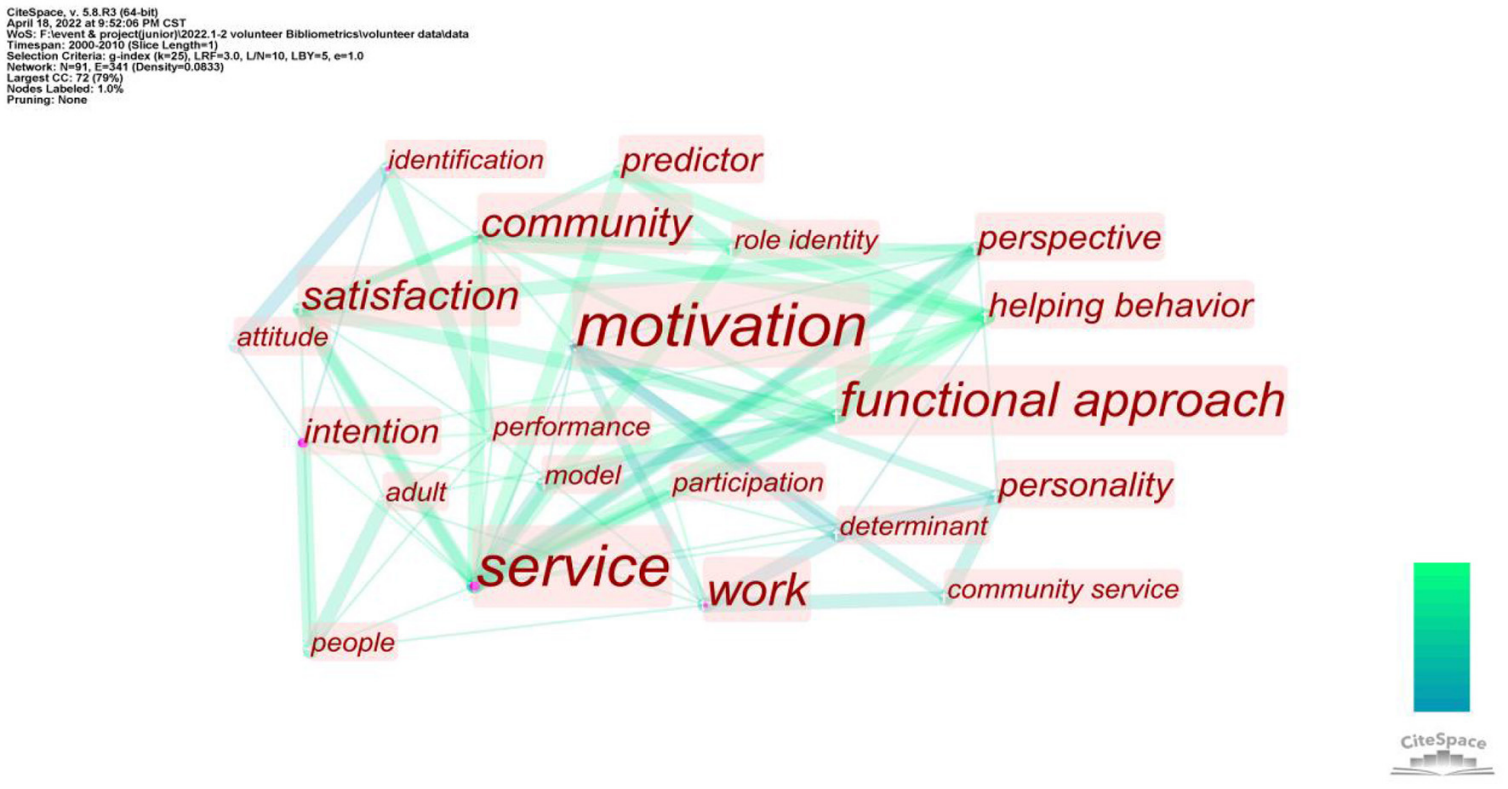
Keyword network between 2000 and 2010 (Threshold for inclusion is a minimum of two occurrences).

As shown in Figure 4, most articles about volunteer motivation from 2000 to 2010 are closely related to Cluster #1 (see Figure 3). The close relation between keywords, such as “community,” “role identity,” “personality,” and Cluster #1 shows that most of the research at this time was guided by a functional approach (Allison et al., 2002; Finkelstein et al., 2005; Houle et al., 2005; Liao-Troth, 2005; Mowen and Sujan, 2005; Kim et al., 2010). Meanwhile, keywords such as “helping behavior,” “service,” and “performance” show that research at this time mainly focused on voluntary activities and motivation without much expansion. Besides, the keyword “satisfaction” indicates that the research at this stage was closely related to Cluster #4 (see Figure 3), which reflects that research on volunteer motivation had paid attention to the important variable of satisfaction (Davis et al., 2003; Finkelstein, 2009).

**Figure 5:**
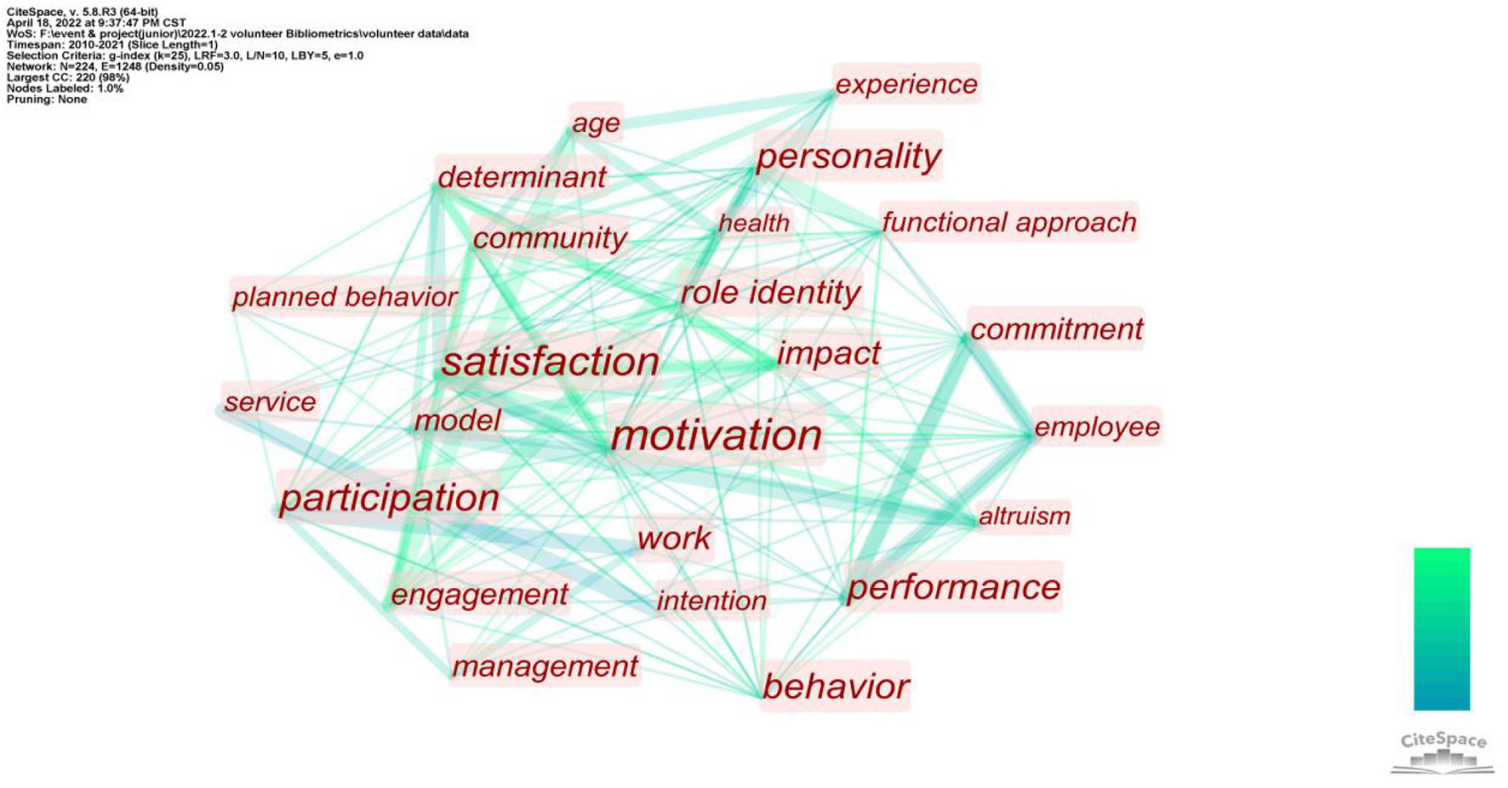
Keyword network between 2011 and 2021 (Threshold for inclusion is a minimum of four occurrences).

As shown in Figure 5, the research topics from 2011 to 2021 show a diversified trend. The presence of keywords such as “planned behavior” and “altruism” indicates that at this time, the theories for the study of volunteer motivation began to gradually increase, and research guided by self-determination theory and planned behavior theory began to appear. The research at this stage began to show a close association between Clusters #2 and #3 (see Figure 3). Although the functional approach was still an important keyword at this stage, its popularity had decreased significantly compared to the proportion of published papers. This research has many subdivisions. For example, some studies began to focus on the influence of past volunteer experience and people's age on volunteer motivation (Kulik, 2017), while others focused on the relationship between volunteer motivation and volunteer management. The keywords “engagement” and “employee” reflect that research on volunteer motivation has expanded to the recruitment and employment of volunteers by relevant organizations (Meneghini et al., 2018). In 2019, after some scholars proposed that past volunteer experience may influence volunteer motivation, volunteer experience gradually became a new focus of volunteer motivation research (Bang et al., 2019).

By comparing Figures 4, 5, it can be seen that although there are some changes in the topics of volunteer motivation research, the focus has not changed greatly: Research is always centered on “motivation,” “satisfaction,” “personality,” “functional approach,” and “intention,” which shows that the main research theme in this field was basically determined before 2010. Most research after 2010 supplements the original research framework and expands it based on original theoretical research (Wicker, 2017).

### Analysis of Burst Keywords

**Figure 6:**
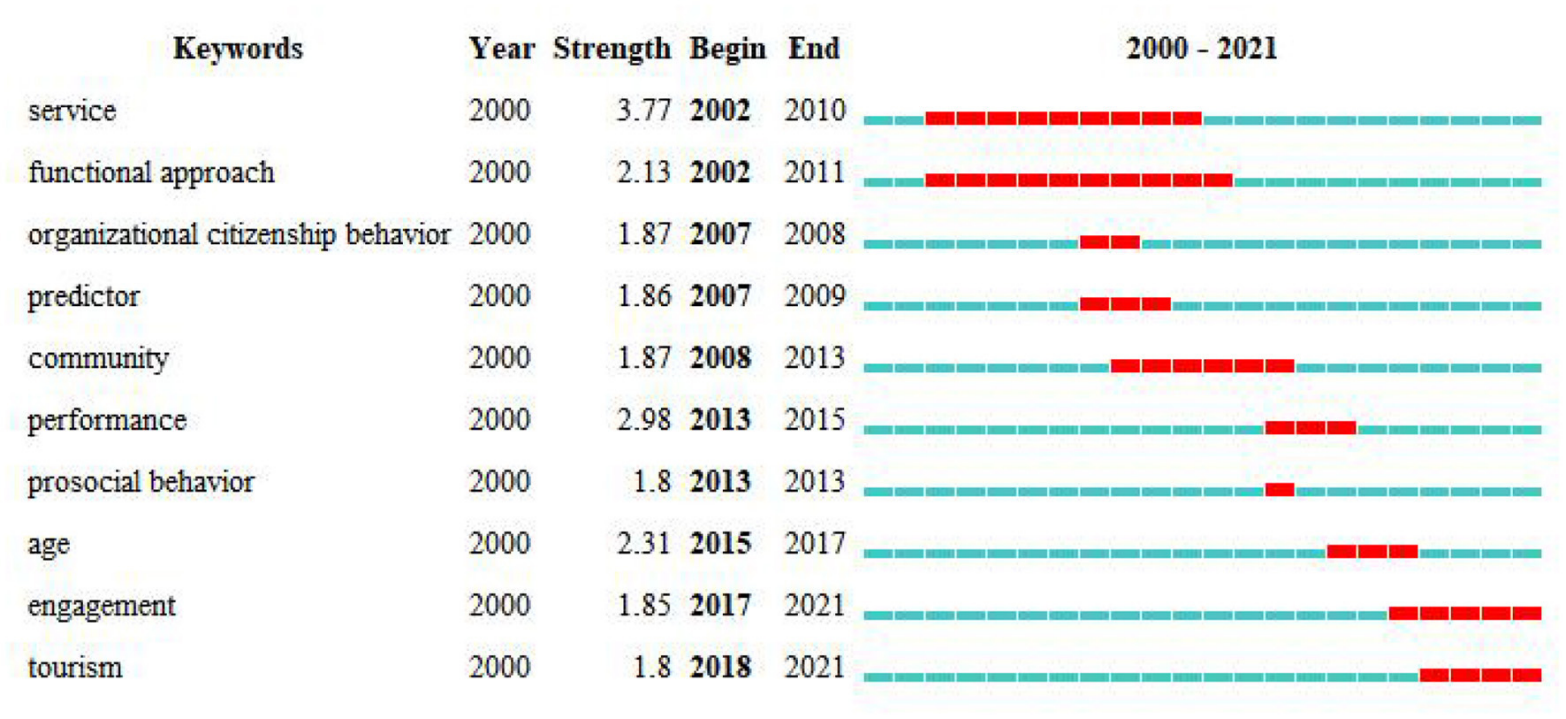
Top 10 keywords with the strongest citation bursts.

The sudden emergence and transformation of keywords can reflect the changes in hotspots in a single research field. Therefore, the burst detection function provided by CiteSpace was used to detect the explosive words with the top 10 explosive intensities (Figure 6).

As shown in Figure 6, in the early twenty-first century, most studies of voluntary motivation regarded volunteer behavior as a service activity or organized citizenship behavior (which appeared as a burst keyword during 2007–2008). Many studies have adopted the functional approach proposed by Clary et al. (1998), the keyword of which showed sustained popularity from 2002 to 2011 (Allison et al., 2002; Finkelstein et al., 2005; Houle et al., 2005; Liao-Troth, 2005; Mowen and Sujan, 2005; Kim et al., 2010). During this period, theory and practice were intertwined. Theory guides many empirical studies, whereas practical research promotes the development of theory.

From 2008 to 2013, volunteer motivation in the community context became a hot topic, and altruism and prosocial behavior attracted the attention of some researchers during this period (Snyder and Omoto, 2008; Hyde and Knowles, 2013). From 2015 to 2017, many studies were conducted on the influence of age on volunteer motivation (Kulik, 2017). These burst keywords reflect that the research topic of volunteer motivation deepened and expanded as the variables of volunteer motivation also increased.

Among the explosive words, there have been two focuses in recent years: the engagement of volunteers in volunteer activities (Meneghini et al., 2018) and volunteers in tourism activities (Paraskevaidis and Andriotis, 2017). Hersberger-Langloh et al. (2021) and other scholars found that volunteers in cultural events tend to engage in a volunteer activity for a longer time. Volunteer tourism activities are rich in forms such as volunteer teaching tourism with Chinese characteristics (Wu et al., 2018) and volunteer motivation and retention research in scenic spots (Ngah et al., 2021).

### Analysis of Chinese and American Volunteer Motivation From a Cross-Cultural Perspective

The cultural background has been verified as a crucial background element affecting volunteer motivation (Aydinli et al., 2016). To learn the influence of Eastern and Western cultures represented by China and the United States on volunteer motivation, 25 Chinese literature, and 38 American literature were selected for analysis. First, we created a high-frequency keyword figure.

**Figure 7:**
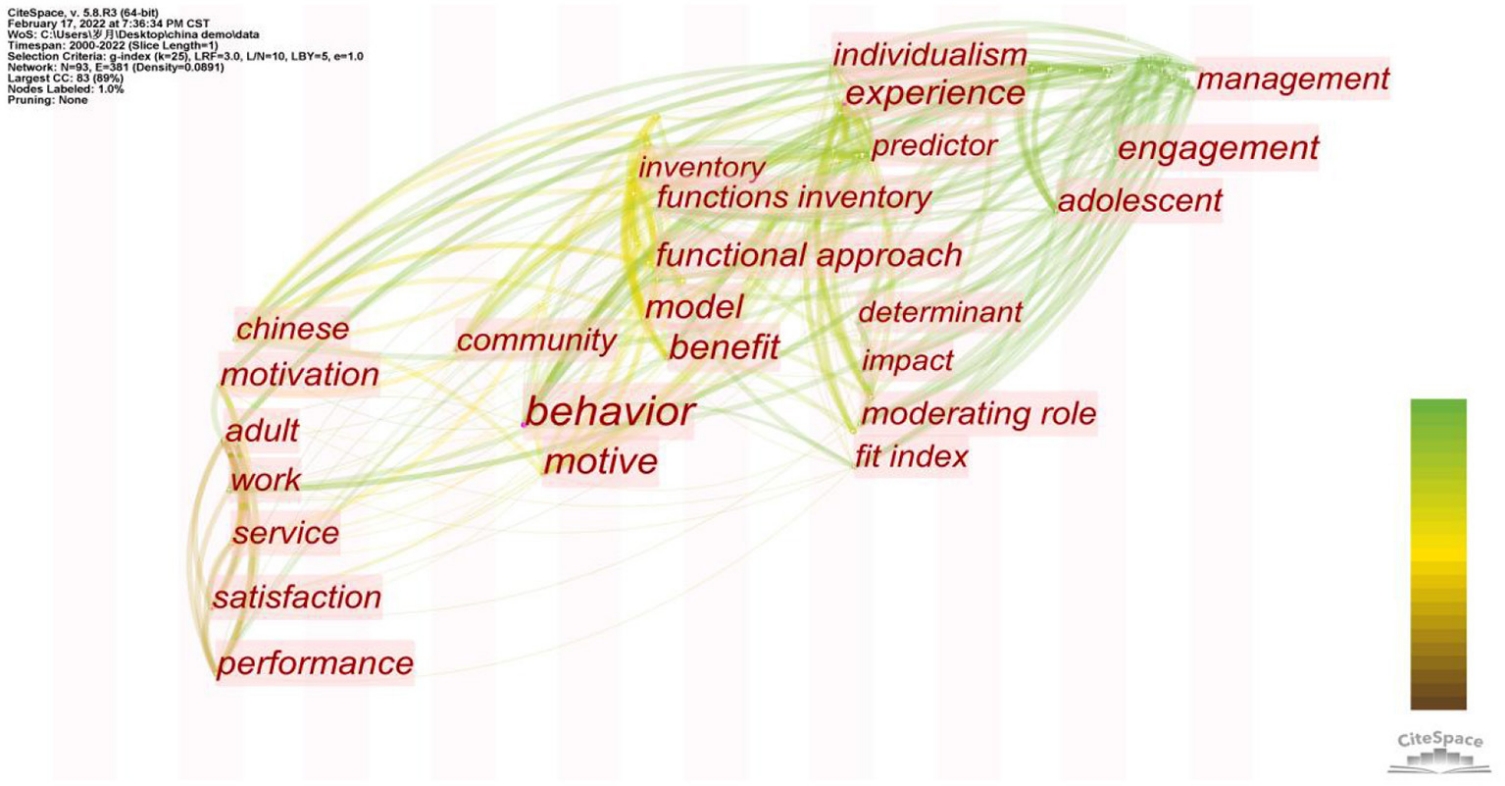
Map of timezone view in Chinese volunteer motivation research.

**Figure 8:**
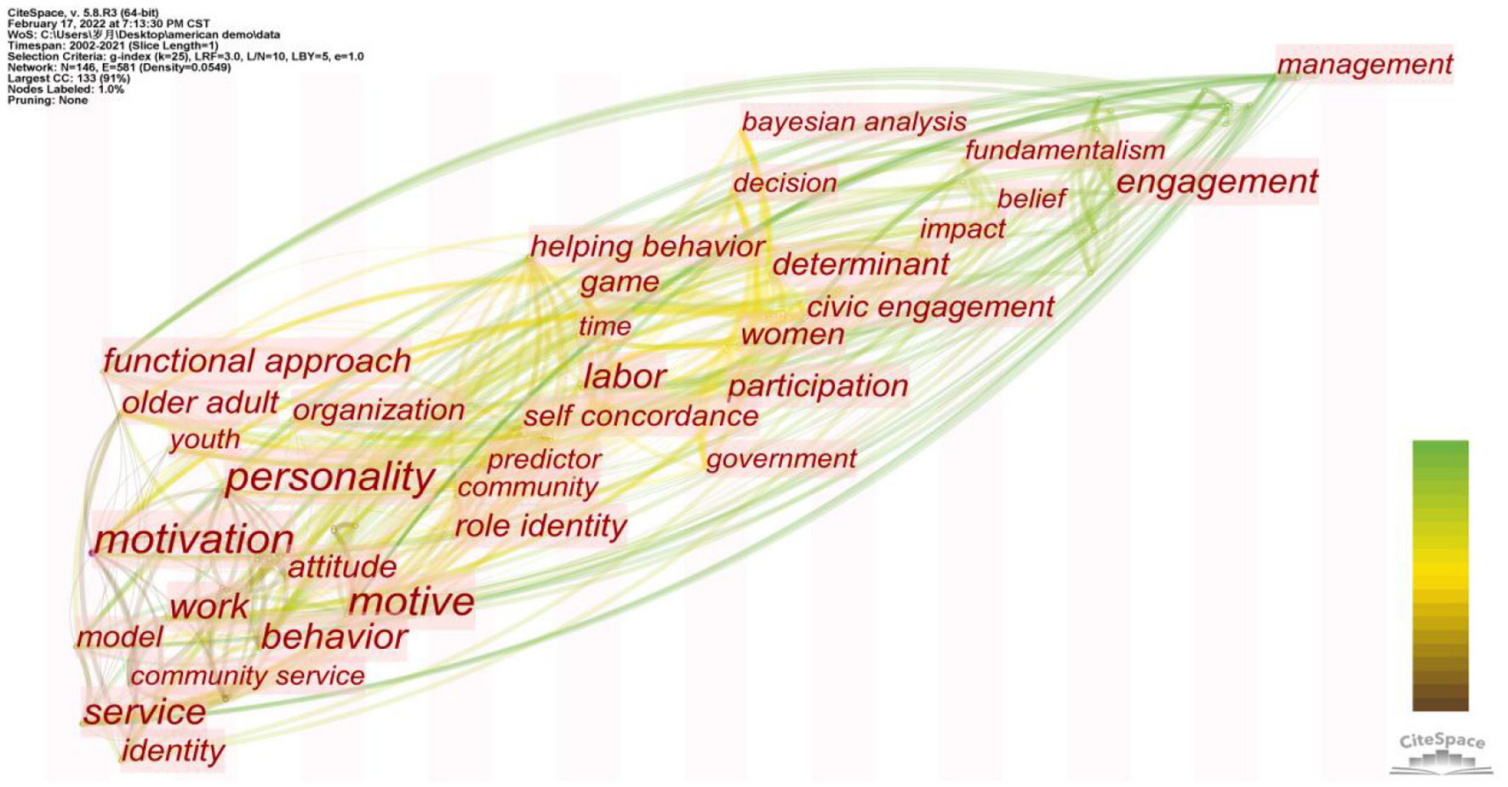
Map of timezone view in American volunteer motivation research.

Table 10 reveals that there are some differences in the research hotspots of volunteers from diverse cultural backgrounds in China and America. Research on volunteers in China focuses more on prosocial motivation (Aydinli et al., 2016), with an emphasis on the contribution of voluntary behavior to society or the community. Research on volunteers in America pays more attention to the personality of volunteers (Finkelstein, 2010), emphasizing their role identity. We used CiteSpace to conduct a visual analysis to better understand the differences and evolutionary trends of volunteer research under diverse cultural backgrounds. The results are shown in Figures 7, 8, respectively.

**Table 10:** High-frequency keywords in different cultural backgrounds.

**Top 10 keywords under**			**Top 10 keywords under**		
**Chinese culture**			**American culture**		
**Rank**	**Keywords**	**Frequency**	**Rank**	**Keywords**	**Frequency**
1	Motivation	20	1	Motivation	34
2	Volunteerism	10	2	Volunteerism	20
3	Behavior	8	3	Personality	12
4	China	7	4	Satisfaction	9
5	Satisfaction	6	5	Functional-Approach	7
6	Prosocial motivation	5	6	Service	7
7	Community	5	7	Work	6
8	Service	4	8	Behavior	5
9	Performance	4	9	Role identity	5
10	Self-determination	4	10	Engagement	4

A comprehensive analysis of Figures 7, 8 show that the study on volunteer motivation derived around 2006 in China, slightly later than that in the United States; in terms of research subjects, college student volunteers are the common focus of volunteer research in China and the United States. The American volunteer study also focused on seniors (Kahana et al., 2013; Okun et al., 2015), but less attention has been paid to China, and only a few studies have been conducted on senior volunteers in Hong Kong (Cheung et al., 2006). From the perspective of the research background, beliefs, and other related keywords are more concerned with religious factors in the study of volunteers in the United States. Since China lacks a broad and unified religious belief, differences in religious culture lead to great disparities in volunteer motivation (Mencken and Fitz, 2013; Cnaan et al., 2017); and with regard to the adopted theory in research, the common occurrence of the keyword in time zone, functional approach, indicates that functional theory has been widely used by Chinese and American researchers with reference to VFI. However, the application of functional theory in the United States is much wider than in China. Through a literature search, 18 of the 38 papers with Americans as the research objects were guided by functional theory, while only 3 of the 25 articles with Chinese as the research objects used functional theory (Lai et al., 2013; Aydinli-Karakulak et al., 2016; Butt et al., 2017). Regarding the time when the theory was used, research on the motivation of Chinese volunteers under the guidance of functional theory began in 2013, while American volunteers were studied earlier. Since Clary initiated the theory in 1998, scholars have applied it successively (Omoto et al., 2000; Allison et al., 2002), with an increase in its application around 2005 (Finkelstein et al., 2005; Houle et al., 2005; Liao-Troth, 2005).

Referring to the variables, keywords such as the community in Figure 7 indicate that the motivation of Chinese volunteers is influenced by collectivism. Value expression, patriotism, pride, and identity are variables observed by researchers (Wang and Wu, 2014) and prosocial motivation is usually prominent (Aydinli et al., 2016). An empirical study of Chinese volunteers highlighted that a stronger national identity often corresponds to more significant volunteer motivation, while national identity significantly influences people's intention to participate in voluntary activities (Lai et al., 2013). Keywords such as personality in Figure 8 indicate that the United States is deeply influenced by the trend of liberalism and focuses on the motivation behind individualism (Finkelstein, 2010). Nevertheless, while concentrating on the motivation for individualism, researchers still focus on collectivism. As can be seen from the figure, although the frequency of keywords such as community service is lower than that of personality, some scholars continue to focus on it (Finkelstein, 2009).

Compared with China, the United States has formed a complete system for the study of volunteer motivation, especially in the study of religious culture. These studies explain the behavior of American volunteers during voluntary service (Ozorak, 2003; Johnston, 2013). Although China has the same high level of citizen volunteer participation as the United States (Rochester et al., 2010), research on volunteer motivation is slightly behind that of the United States in terms of time and is influenced by its culture. Moreover, past studies have often neglected some motivations of individualism, and from the development trend shown in the time zone map, research on Chinese scholars is progressing with the research development trend. It could be perceived from the two keywords, engagement, and management, that recently, China and the US have tended to share similar hotspots.

## Conclusions, Implications, and Prospect

### Conclusions

Volunteer motivation, as a topic that evolves with the times, still enjoys popularity in various disciplines such as psychology, management, and sociology. This study analyzes relative studies on volunteer motivation from 2000 to 2021 with VOSviewer and CiteSpace, and systematically reviews its development trend using a bibliometric method. Meanwhile, this study discusses and analyzes the core authors, national institutions with high productivity, key journals, and keyword clusters, draws knowledge maps, and summarizes the existing theoretical and empirical research on volunteer motivation. Based on the bibliometric analysis, the following conclusions were drawn.

(1) Research on volunteer motivation seems to be empirical. In recent years, the selected backgrounds in research have gradually diversified, from major sports events in the past to various activities including the World Expo (Lee et al., 2014; Wang and Wu, 2014) and international conferences (Qi et al., 2019). Simultaneously, cross-border comparative research has begun to receive attention (Allik and Realo, 2004; Aydinli et al., 2016). Being sensitive to significant social reforms, the change in society, such as the spread of COVID-19, serves as an important driver in promoting its development and external extension (Trautwein et al., 2020; Kifle Mekonen and Adarkwah, 2021).
(2) Diverse cultural backgrounds form different motivations for volunteering. Some motivations have universality (Aydinli et al., 2016), while those deeply affected by cultural backgrounds tend to play various roles in distinct social environments (Aydinli et al., 2013). Consider the comparison between China and the United States as an example. Among the motivations of Chinese volunteers, collectivism seems to be dominant (Wang and Wu, 2014; Guo et al., 2021); because of cultural differences, American volunteers are attentive to the motivation factors of collectivism, but also focus on individualism (Mencken and Fitz, 2013; Cnaan et al., 2017; Yamashita et al., 2019).
(3) Those authors who have published more than or equal to three articles in this research field in the last 20 years are regarded as high-productivity authors. Twelve authors accounted for 27.6% of the total number of published papers. In terms of countries and organizations, the most active countries in this field are mainly Europe and North America. Regarding published articles, the United States ranked first in this field, followed by China and Australia.
(4) To analyze publishing journals, the number of published articles in this field in *Voluntas* and the *Non-profit and Voluntary Sector Quarterly* takes the first two positions. The development of open access, a driving force in this field with a recent, rapid increase in published articles, effectively aroused study enthusiasm. Further analysis of journals found that those related to management, non-profit organizations, volunteer research, and social psychology enjoyed the highest number of citations. The main purpose of citing management journals is to obtain theoretical support (Dwyer et al., 2013; Güntert and Wehner, 2015) while citing psychological journals provides evidence for scale design (Bang et al., 2019).
(5) The co-occurrence analysis and cluster analysis of keywords reveal that self-determination theory (Finkelstein, 2009; Güntert and Wehner, 2015; Van Schie et al., 2015) and the theory of planned behavior (Warburton and Terry, 2000; Greenslade and White, 2005; Hyde and Knowles, 2013; White et al., 2017; Almas et al., 2020) are the two most frequently applied. An evolution analysis revealed that a mature research system has been developed in this field. After 2010, the two trends of research were the investigation of the connotation of volunteer motivation variables and the increase of research subjects. Overall, the scope of this research field is expanding.

### Practical Implications

This study clarified the quantization of published articles on volunteer motivation through bibliometric analysis. Based on this conclusion, core articles were selected to read, analyze, and review, which shows the development overview, research focus, and evolution trend of this field. They provide theoretical support for the formulation of volunteer recruitment strategies (Wicker, 2017). The practical value of this study is as follows:

(1) This study explores the theoretical basis (self-determination theory, theory of planned behavior and function theory) and research methods (empirical research methods such as structural equation model) of volunteer motivation research topics and summarizes the commonly used scales and their variables in this field. Furthermore, this study constructs a clear framework for the existing studies in this field, clarifying the development process.
(2) This study analyzed the clusters of high-frequency keywords and their evolution trends. It illustrates the focus and hotspots of this field. Moreover, recently, it discovered some new hot spots in volunteer motivation (volunteer engagement and the relationship between volunteer motivation and management). Thus, it provides a decision-making reference for the selection of research topics.
(3) This study analyzed the motivation of volunteers from diverse cultural backgrounds represented by China and the United States. It was found that there are distinctive differences that can provide a basis for volunteer organizations to recruit volunteers from various cultures.
(4) Through the analysis of the number of articles, this study identified two core journals in the field of volunteer motivation research (*Voluntas* and *Non-profit and Voluntary Sector Quarterly*) and found several comprehensive open access journals which published many articles in this field (*Sustainability* and *Journal of Social Service Research*). To a certain degree, the analysis provides guidance for scholars to choose the appropriate journal when trying to publish articles on this topic.

### Limitations and Future Research

This study had some limitations owing to objective factors. First, bibliometric analysis software has a high data requirement. Therefore, this study only selected articles in SSCI from the core collection of the Web of Science to ensure the quality and integrity of the collected papers. We excluded other databases, conference papers, and comments; therefore, we may have neglected some scientific research and unique opinions. Although this study obtained objective quantitative data with professional software for bibliometric analysis, the analysis and interpretation of data would inevitably be subjective. Therefore, the impact of subjectivity on the data analysis is unlikely to be completely avoided.

In sum, future studies will widen the scope of articles and understand research trends and frontier hotspots. Furthermore, we intend to actively communicate with scholars who are experts in the field of volunteer motivation to acquire objective and frontier views to minimize the negative impact of personal subjectivity on research analysis.

This study applied systematic and scientific research methods to summarize the main research topics in volunteer motivation research. It was found that changes in research theories have played an important role in promoting research on volunteer motivation. Therefore, it can be reasonably predicted that more research theories will be applied to boost its prosperity and development. However, because of the limited space, there are still some aspects of this study remaining to be explored. For example, we didn't classify and quantify the types of volunteer activities, which should be refined to gain more comprehensive quantitative information, so as to get more rigorous conclusions. Meanwhile, although this study made a comparative analysis of volunteer research in China and in the US, the coverage is still not wide enough. We suggest that future studies should cover the differences in volunteer motivation in various countries and regions, and analyze its mechanism to reveal the influence of different cultures on people's volunteer motivation behavior.

## Data Availability Statement

The original contributions presented in the study are included in the article/supplementary material, further inquiries can be directed to the corresponding author/s.

## Author Contributions

JC, CW, and YT contributed equally to the conception of the idea, implementing and analyzing the experimental results, and writing the manuscript. All authors have read and agreed to the published version of the manuscript.

## Funding

This research was supported by the National Social Science Fund of China (21CSH049).

## Conflict of Interest

The authors declare that the research was conducted in the absence of any commercial or financial relationships that could be construed as a potential conflict of interest.

## Publisher's Note

All claims expressed in this article are solely those of the authors and do not necessarily represent those of their affiliated organizations, or those of the publisher, the editors and the reviewers. Any product that may be evaluated in this article, or claim that may be made by its manufacturer, is not guaranteed or endorsed by the publisher.
